# Supplementary material for: Linear time complexity de novo long read genome assembly with GoldRush
Source: Nat Commun. 2023 May 22;14:2906. doi: 10.1038/s41467-023-38716-x (PMC10202940; doi:10.1038/s41467-023-38716-x)
Supplement: Supplementary file 1 — Supplementary Information [file 41467_2023_38716_MOESM1_ESM.pdf]

Supplementary Information for:
Linear time complexity *de novo* long read genome assembly with GoldRush

Johnathan Wong, Lauren Coombe, Vladimir Nikolić, Emily Zhang, Ka Ming Nip, Puneet Sidhu, René L Warren, and Inanç Birol

These authors contributed equally: Johnathan Wong, Lauren Coombe

Table of Contents

Supplementary Fig. 1: Flowchart of the GoldRush assembly pipeline. 5

Supplementary Fig. 2: Duplicated gene counts in multiple copies in genome assemblies of the NA24385 human cell line, generated by GoldRush and comparator tools. 6

Supplementary Fig. 3: Spectra-cn plot comparisons of the NA24385 genome assemblies. .. 7

Supplementary Fig. 4: GoldPolish thread optimization schematic. 8

Supplementary Fig. 5: Ideogram plots showing the contiguity of the three GoldRush human genome assemblies (NA24385, HG01243, and HG02055). 9

Supplementary Fig. 6: Example of logic for inserting read signatures to the miBf data structure and golden path.. 10

Supplementary Fig. 7: Example of querying the miBf with a new read. 11

Supplementary Fig. 8: Improving the accuracy of the tile's best hit.................................. 12

Supplementary Fig. 9: Example of the three different outcomes when querying a read. 13

Supplementary Fig. 10: Schematic describing the GoldPolish base error correction protocol. 14

Supplementary Fig. 11: The overlap detection and resolution feature of ntLink................ 15

Supplementary Fig. 12: The gap-filling feature of ntLink. 16

Supplementary Fig. 13: Contiguity and correctness results of assembling long reads from the human cell line NA24385 with GoldRush, sweeping on the GoldChain *k* and *w* parameters... 17

Supplementary Fig. 14: Contiguity and correctness results of assembling long reads from the human cell line HG01243 using GoldRush, sweeping on the GoldChain *k* and *w* parameters... 18

Supplementary Fig. 15: Contiguity and correctness results of assembling long reads from the human cell line HG02055 using GoldRush, sweeping on the GoldChain *k* and *w* parameters. 19

Supplementary Fig. 16: Contiguity and correctness results of assembling long reads from *O. sativa* using GoldRush, sweeping on the GoldChain *k* and *w* parameters. 20

Supplementary Fig. 17: Contiguity and correctness results of assembling long reads from *S. lycopersicum* using GoldRush, sweeping on the GoldChain *k* and *w* parameters. 21

Supplementary Table 1: Contiguity and correctness statistics of human cell line NA24385 genome assemblies generated by GoldRush and the comparator tools. 22

Supplementary Table 2: Contiguity and correctness statistics of human cell line HG01243 genome assemblies generated by GoldRush and the comparator tools. 22

Supplementary Table 3: Contiguity and correctness statistics of human cell line HG02055 genome assemblies generated by GoldRush and the comparator tools. 23

Supplementary Table 4: Contiguity and correctness statistics of *O. sativa* genome assemblies generated by GoldRush and the comparator tools. 23

Supplementary Table 5: Contiguity and correctness statistics of *S. lycopersicum* genome assemblies generated by GoldRush and the comparator tools. 23

Supplementary Table 6: ONT long read sequencing reads used for genome assembly benchmarks. 24

Supplementary Table 7: Resource usage of GoldRush and the comparator tools for the genome assembly of the human cell line NA24385. 24

Supplementary Table 8: Resource usage of GoldRush and the comparator tools for the genome assembly of the human cell line HG01243. 25

Supplementary Table 9: Resource usage of GoldRush and the comparator tools for the genome assembly of the human cell line HG02055. 25

Supplementary Table 10: Resource usage of GoldRush and the comparator tools for the genome assembly of the *O. sativa* dataset. 25

Supplementary Table 11: Resource usage of GoldRush and the comparator tools for the genome assembly of the *S. lycopersicum* dataset. 26

Supplementary Table 12: Contiguity and correctness statistics of human cell line NA24385 genome assembly generated by GoldRush at different stages. 26

Supplementary Table 13: BUSCO statistics for assemblies of human cell line NA24385 generated by GoldRush and the comparator tools. 27

Supplementary Table 14: Counts of duplicated genes found in multiple copies in genome assemblies of the NA24385 human cell line, generated by GoldRush and comparator tools. 27

Supplementary Table 15: Contiguity and correctness statistics of human cell line HG01243 genome assembly generated by GoldRush at different stages. 28

Supplementary Table 16: Contiguity and correctness statistics of human cell line HG02055 genome assembly generated by GoldRush at different stages. 28

Supplementary Table 17: BUSCO statistics for assemblies of human cell line HG01243 generated by GoldRush and the comparator tools. 29

Supplementary Table 18: BUSCO statistics for assemblies of human cell line HG02055 generated by GoldRush and the comparator tools. 29

Supplementary Table 19: Resource usage breakdown of each GoldRush stage for the genome assembly of human cell line NA24385 using Racon instead of GoldPolish. 30

Supplementary Table 20: Resource usage breakdown of each GoldRush stage for the genome assembly of human cell line NA24385. 30

Supplementary Table 21: Contiguity and correctness statistics of the GoldRush genome assembly of the human cell line NA24385 using Racon instead of GoldPolish at different stages. 31

Supplementary Table 22: BUSCO statistics for assemblies of human cell line NA24385 generated by GoldRush using Racon instead of GoldPolish. 31

Supplementary Table 23: Reference-free QV estimation statistics using Merqury for NA24385 GoldRush genome assemblies with GoldPolish or Racon. 32

Supplementary Table 24: Reference-free QV estimation statistics using Merqury for non-repetitive or repetitive regions of the NA24385 GoldRush genome assembly using GoldPolish for the polishing step. 32

Supplementary Table 25: Reference-free QV estimation statistics using Merqury for non-repetitive or repetitive regions of the NA24385 GoldRush genome assembly using Racon for the polishing step. 32

Supplementary Table 26: Resource usage breakdown of each GoldRush stage for the genome assembly of the human cell line HG01243. 33

Supplementary Table 27: Resource usage breakdown of each GoldRush stage for the genome assembly of the human cell line HG02055. 33

Supplementary Table 28: Run time (wall clock) breakdown of each ntLink round within the GoldChain stage of GoldRush, NA24385 genome assembly. 34

Supplementary Table 29: Optimized parameters used for the GoldRush genome assemblies. 34

Supplementary Table 30: Reference genome builds used for QUAST assembly analysis. . 35

Supplementary Table 31: NA24385 cell line data used for Merqury base quality analysis. 35

Supplementary Table 32: Reference genome used for gene duplication analysis. 35

Supplementary Table 33: cDNA sequences used for gene duplication analysis. 35

Supplementary Table 34: Run time (wall clock) breakdown for each step of the first round of ntLink within the GoldChain stage of GoldRush, NA24385 genome assembly. 36

Supplementary Method 1: Improving the accuracy of the best hits for tiles. 37

Supplementary Note 1: Time complexity analysis of GoldRush. 38

Supplementary References 39

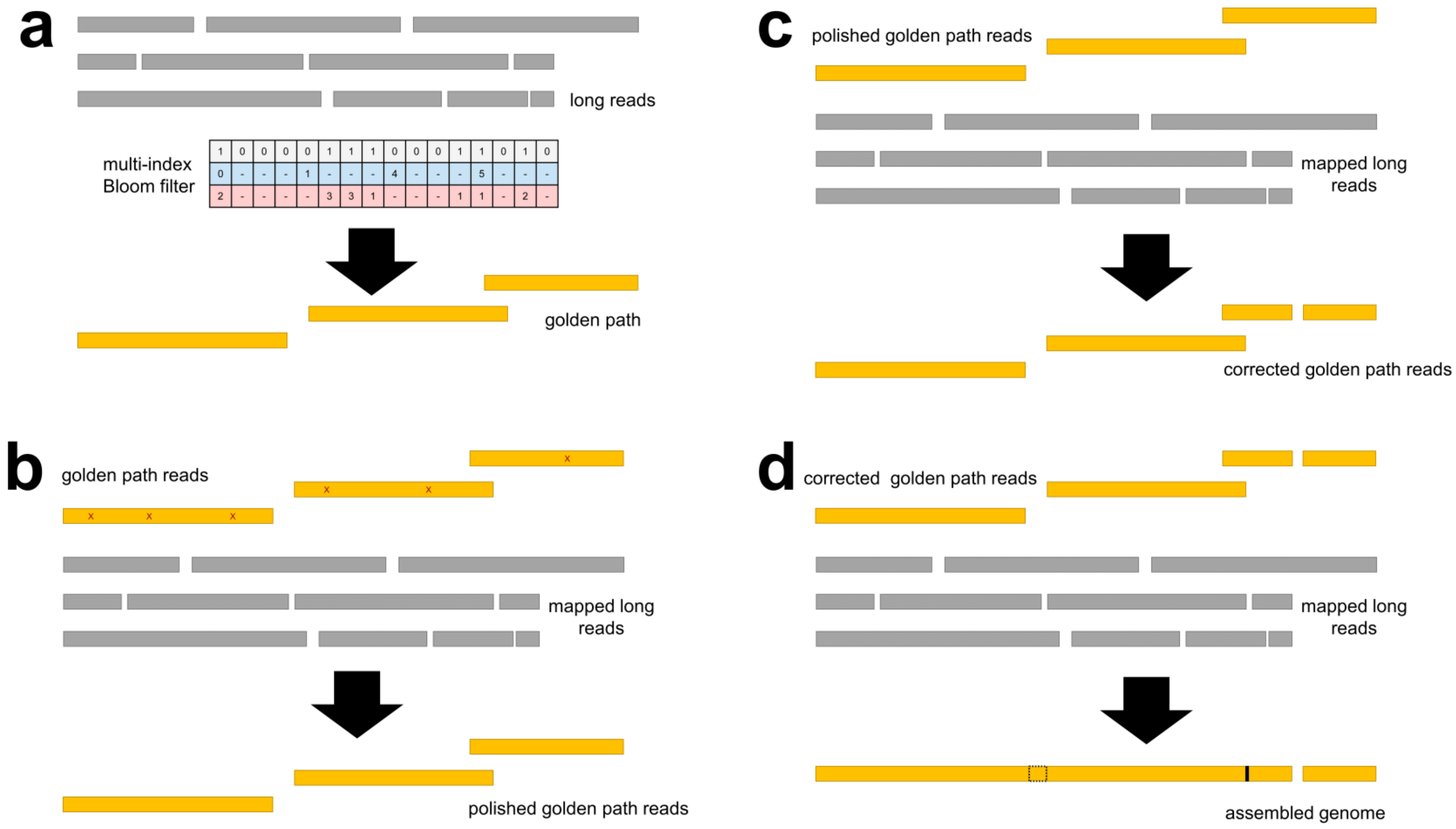

Supplementary Fig. 1: Flowchart of the GoldRush assembly pipeline. In **a**, GoldPath queries the long reads against the multi-index Bloom filter to generate the golden path, a ~1X representation of the underlying genome. The golden path (errors shown as red "X"s) is then polished by GoldPolish in **b**, and corrected with Tigmint-long in **c**. Finally, GoldChain scaffolds (gap filling shown in dotted box and trimming shown in black) the polished and corrected golden path to generate the final genome assembly in **d**.

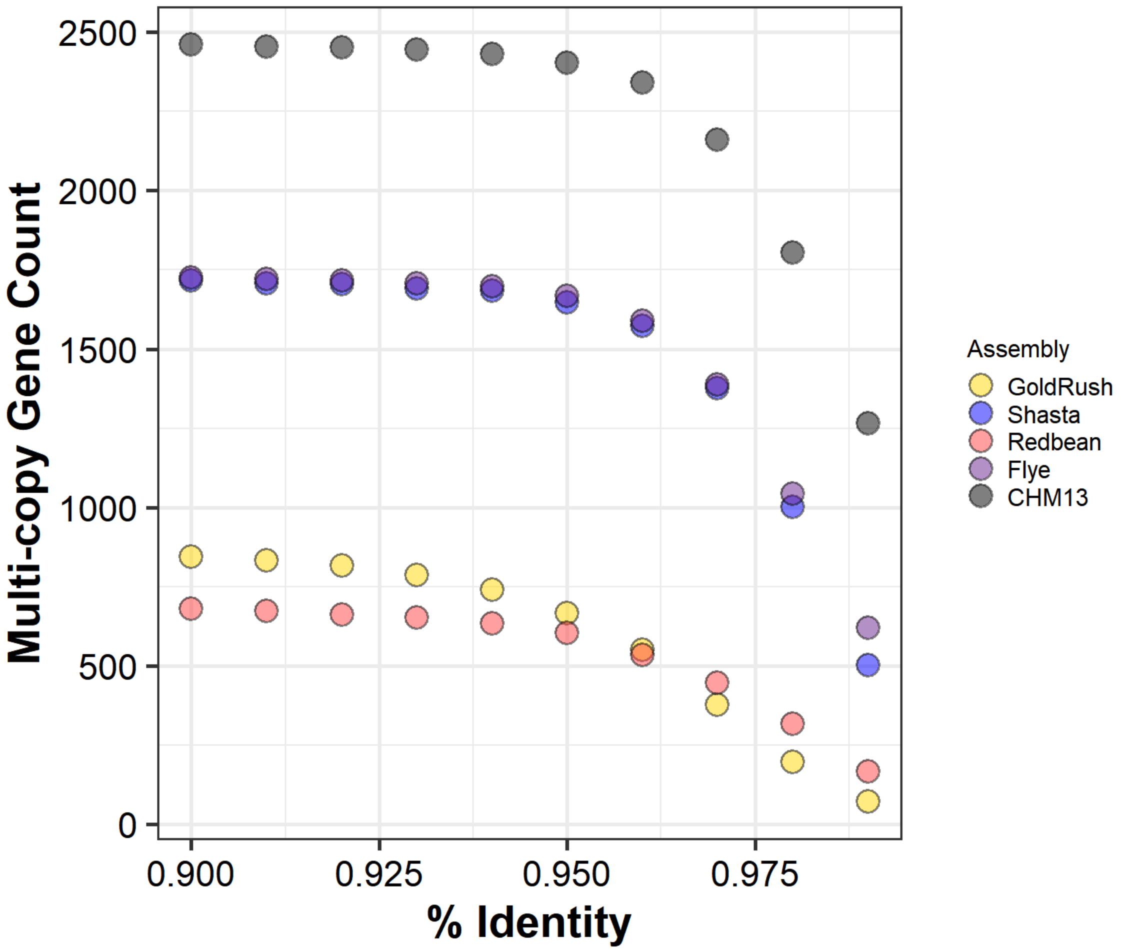

Supplementary Fig. 2: Duplicated gene counts in multiple copies in genome assemblies of the NA24385 human cell line, generated by GoldRush and comparator tools. The asmgene utility in minimap2[1] was run using an identity of [0.90 to .99] and a coverage of 0.99 using all GRCh38 Ensembl[2] cDNA sequences (release 87). The T2T-CHM13[3] (v1.1) genome assembly is used as a point of reference.

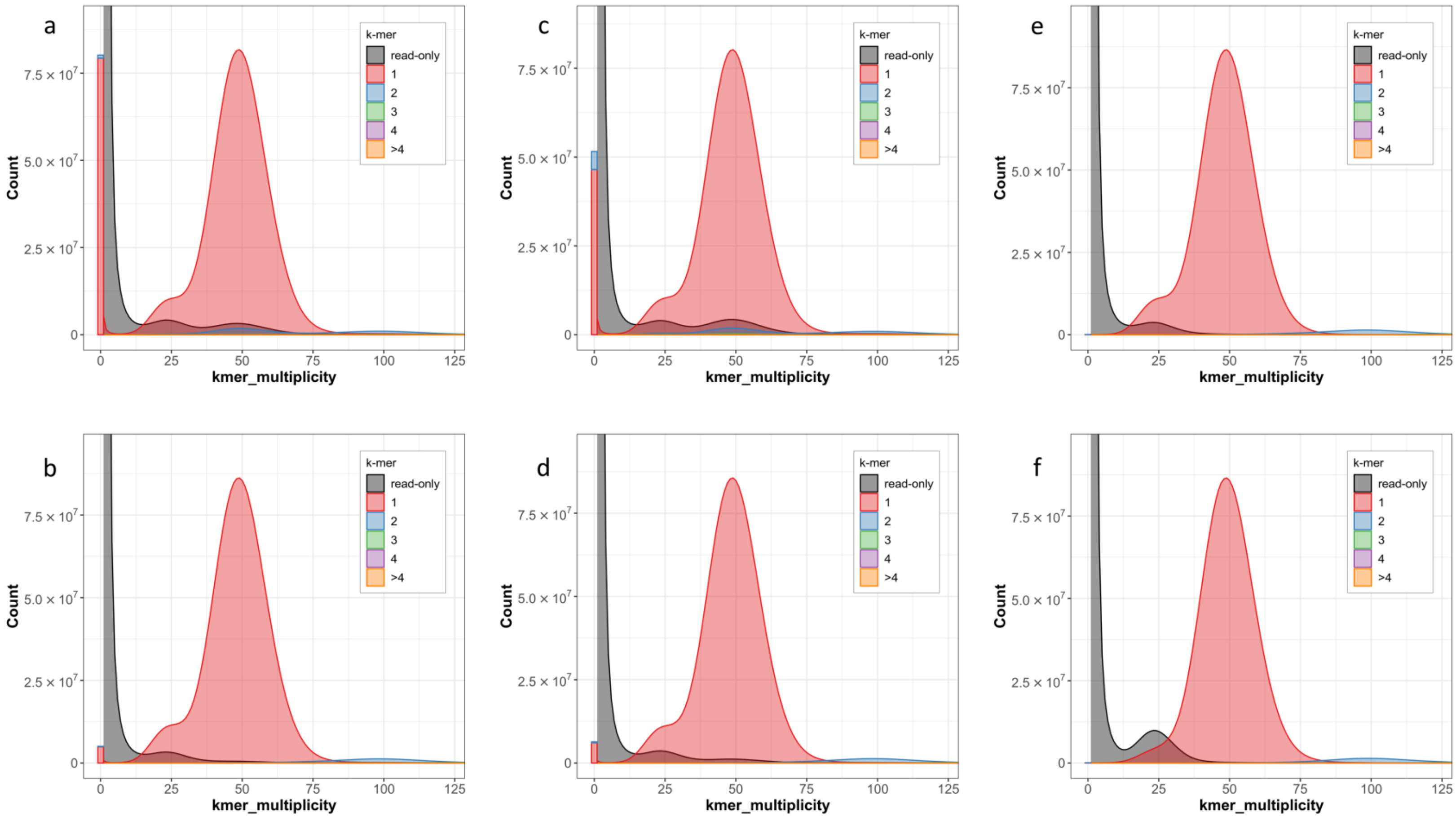

Supplementary Fig. 3: Spectra-cn plot comparisons of the NA24385 genome assemblies. The spectra-cn plot was generated by Merqury using the **a** GoldRush, **b** Flye, **c** Redbean, **d** Shasta, and a reference-grade **e** maternal and **f** paternal NA24385 genome assembly with a Meryl *k*-mer database[4]. The Meryl database was generated using $k = 21$ with NA24835 Illumina short reads (SRR11321732) from the Genome in a Bottle Consortium[5].

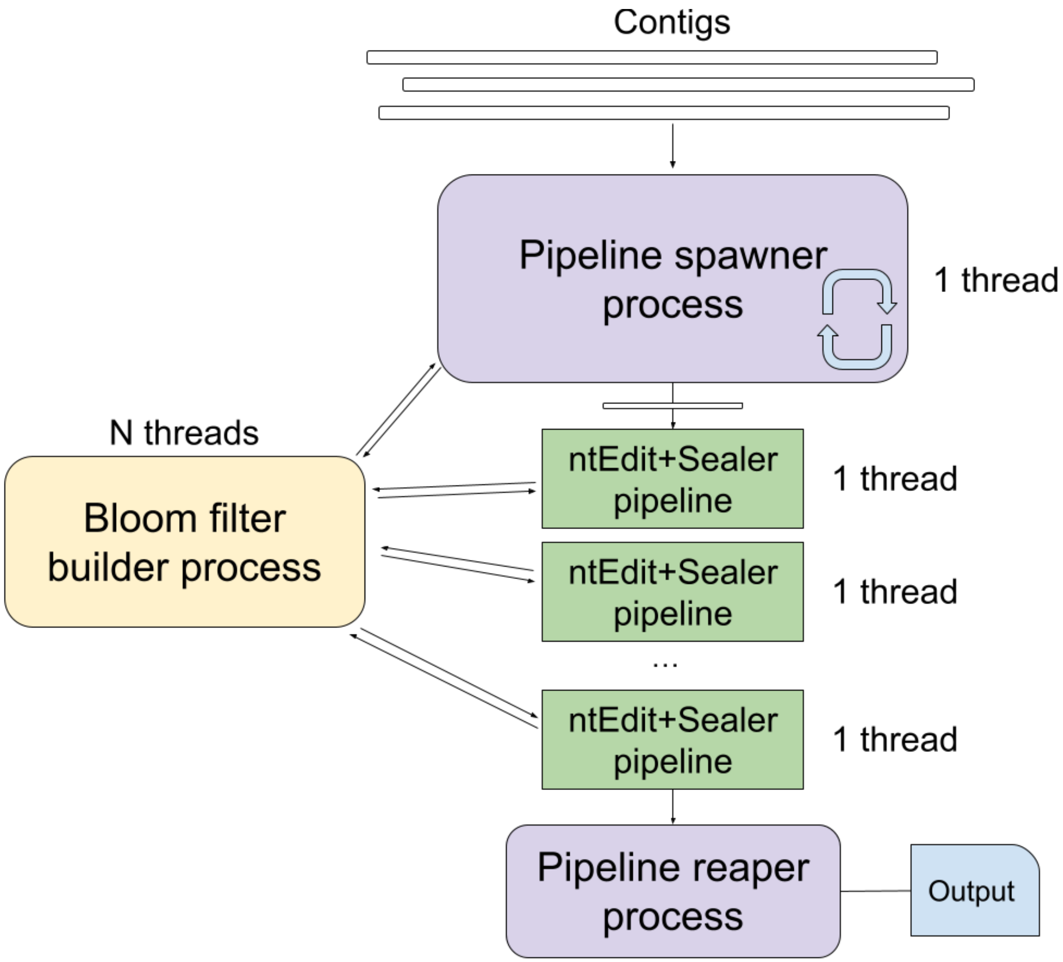

Supplementary Fig. 4: GoldPolish thread optimization schematic. GoldPolish coordinates a set of processes in order to maximize concurrency. A pipeline spawner process launches an instance of ntEdit+Sealer[6] per goldtig to polish, up to a specified limit at a time. The Bloom filter builder process continually builds the required Bloom filters for the subsequent polishing pipelines. Finally, a reaper process collects the results of each polishing process in the order they were started and consolidates the polished sequences to a single output fasta file.

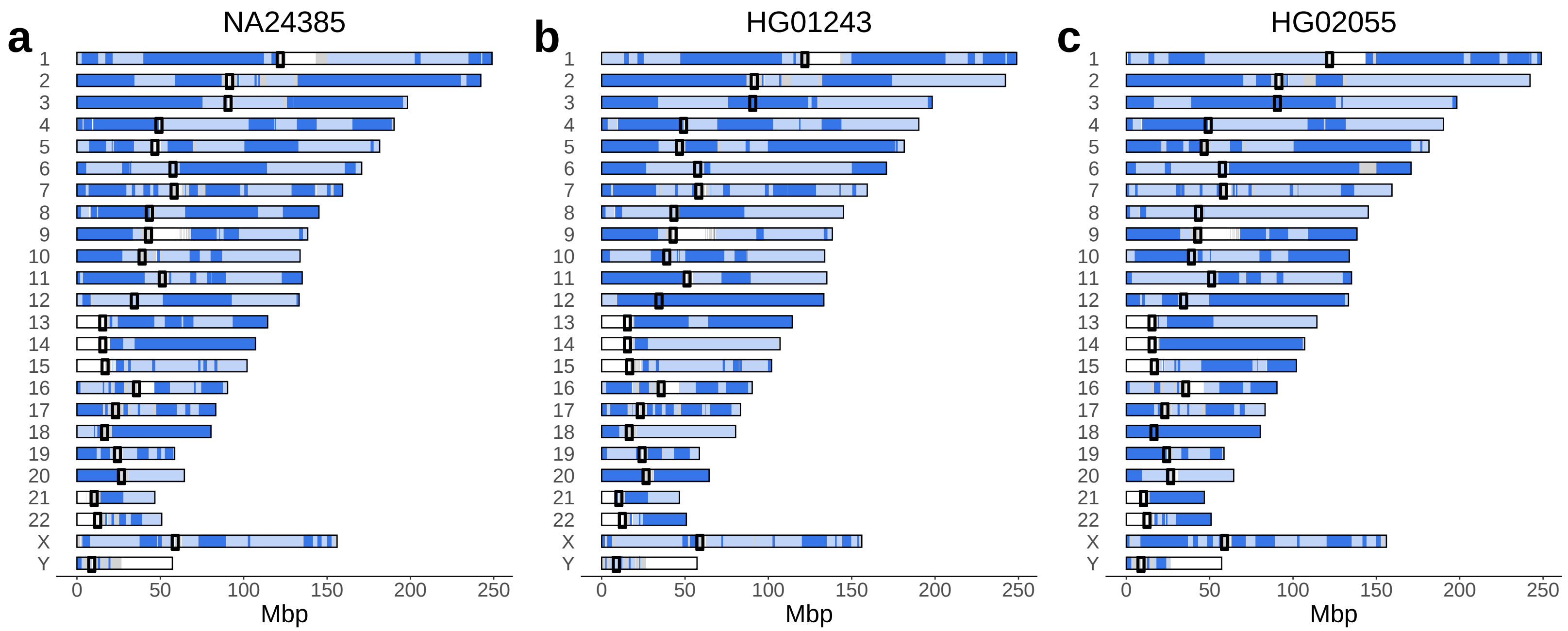

Supplementary Fig. 5: Ideogram plots showing the contiguity of the three GoldRush human genome assemblies (NA24385, HG01243, and HG02055). The ideogram is created by selecting sequences that are least the NG90 length, aligning the sequences to the human reference genome (GRCh38) using minimap2[1], and plotting the resulting alignment blocks. White regions are Ns in the reference genome, and grey regions have no mapped sequences. The aligned scaffolds are represented with alternating shades of blue. For all three human cell lines, GoldRush assembled multiple full chromosome arms. In **a** and **b**, GoldRush assembled both chromosomal arms of chromosome 20 and one chromosomal arm of chromosome 18. In **c**, GoldRush assembled chromosome 18 in one piece.

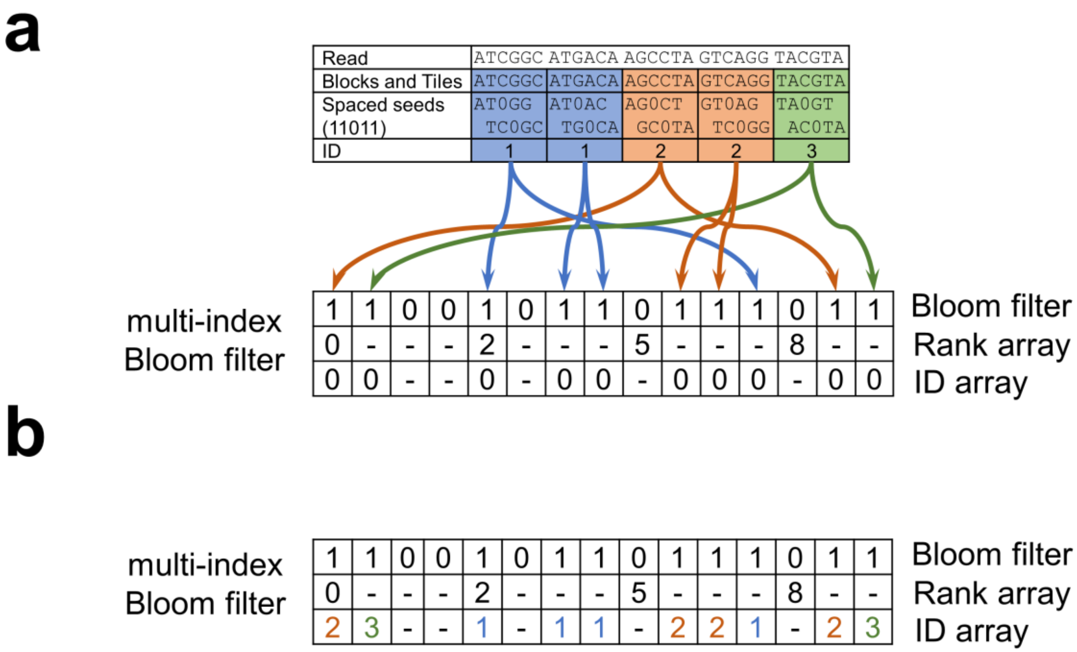

Supplementary Fig. 6: Example of logic for inserting read signatures to the miBf data structure and golden path. A read is being inserted into the miBf[7] in **a**. The read is inserted using *t* (length of tile) = 6, *b* (number of tiles in a block) = 2, *h* (number of spaced seed patterns) = 1, with a spaced seed of pattern of 11011. The read is first divided into tiles of length *t*. This is represented by each smaller cell. Cells that belong to the same block are given the same colour. The blue, orange, and green cells are associated with the IDs 1, 2, 3, respectively. The sequence in each tile is hashed using the spaced seed pattern and inserted into the miBf. **b** shows the changes in the miBf after insertion. The colors of the IDs in the ID array in **a** correspond to the blocks they are derived from in **a**.

a

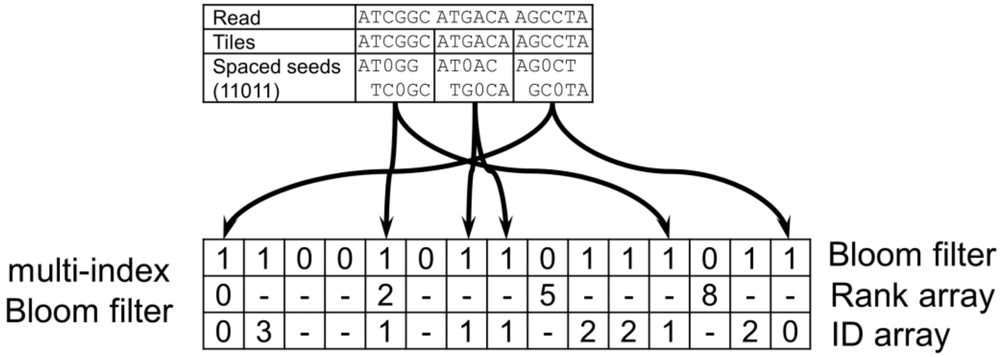

b

Read	ATCGGC ATGACA AGCCTA		
Tiles	ATCGGC	ATGACA	AGCCTA
Spaced seeds (11011)	AT0GG TC0GC	AT0AC TG0CA	AG0CT GC0TA
ID: counts tally	1:2	1:2	
Associated ID	1	1	0
Tile classification	true	true	false

Supplementary Fig. 7: Example of querying the miBf with a new read. A read is being queried against the miBf[7] in **a**. The read is queried using t (length of tile) = 6, and x (hit threshold for a tile to be assigned) = 1, with a spaced seed of pattern of 11011. The read is first divided into tiles of length t. This is represented by each smaller cell. The sequence in each tile is hashed using the spaced seed pattern and queried against the miBf. **b** shows the results of each cell's query. Both the first and the second cell have two hits to ID 1. GoldPath will then associate these two tiles with an ID of 1, and assess these two tiles as true (assigned) because the number of hits to ID 1 is greater than x, which is 1. On the other hand, there are no hits for the third tile. GoldPath will associate the tile with an ID of 0, and assess it as false (unassigned).

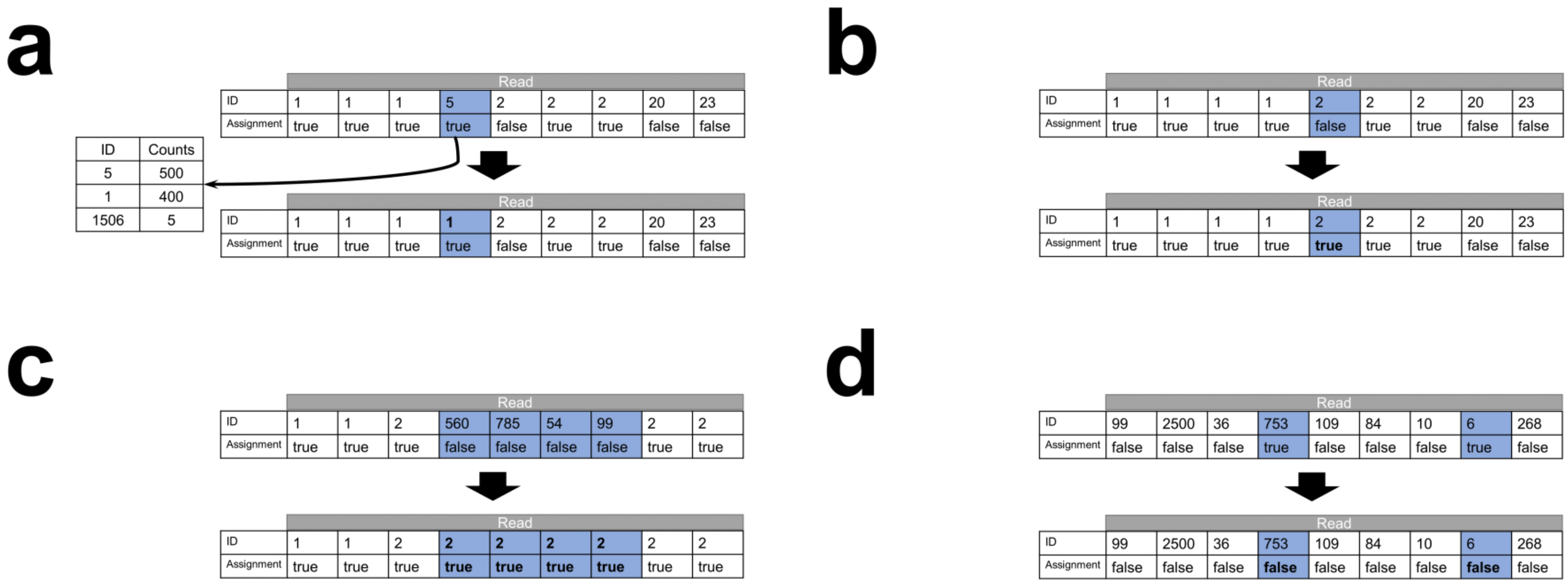

Supplementary Fig. 8: Improving the accuracy of the tile's best hit. In **a**, GoldPath identifies the tile with an associated ID of 5 for improvement (shaded in blue). The neighbour IDs are 1 and 2, and there is an entry in the ID-to-count tables with an ID of 1, so the tile's ID is set to 1. In **b**, GoldPath identifies the unassigned tile with an associated of ID of 2 for improvement (shaded in blue). The adjacent tiles have the same ID or 1 smaller than the current tile, so the current tile is set to assigned. In **c**, a stretch of unassigned tiles is flanked by assigned tiles with the same ID (shaded in blue). The stretch of unassigned tiles is then set to assigned and have their IDs changed to 2. In **d**, there are isolated assigned tiles flanked by unassigned tiles (shaded in blue). These are likely tiles assigned due to false positive hits, so these tiles are set to unassigned.

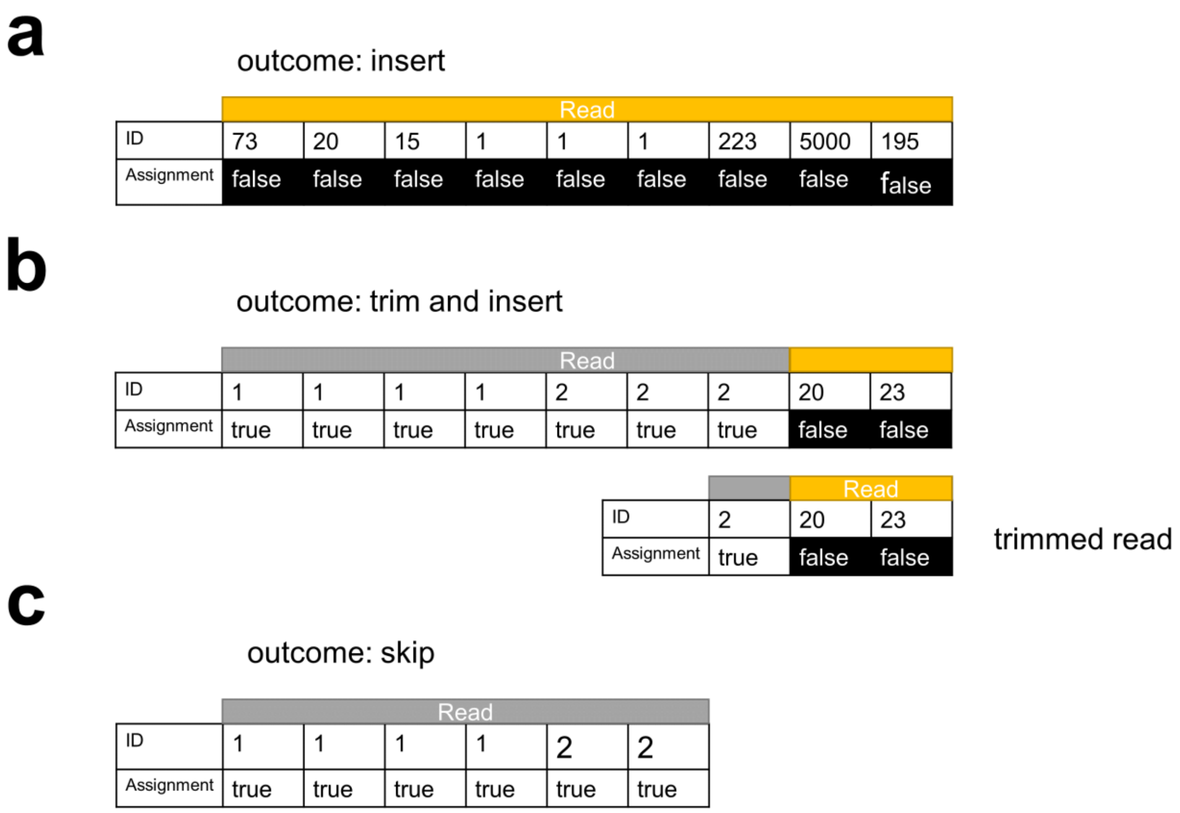

Supplementary Fig. 9: Example of the three different outcomes when querying a read. In **a**, every tile of the read is unassigned (false) which means the genomic locus the read is sequenced from is not captured in the miBf[7], so we insert the read into the miBf. In **b**, the read has a mixture of assigned and unassigned tiles. In this case, we trim the read and retain one tile overhang from the unassigned region. The trimmed read is inserted into the miBf. In **c**, every tile in the read is classified as assigned. Therefore, the genomic locus the read represents is already captured in the miBf, and the read is skipped.

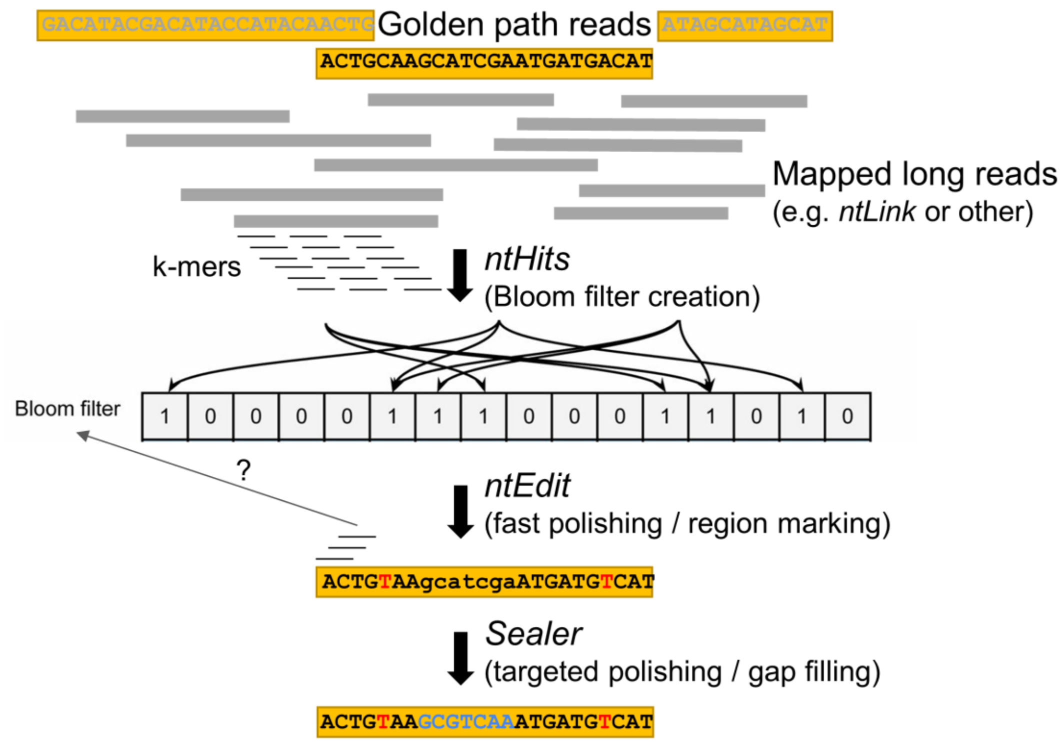

Supplementary Fig. 10: Schematic describing the GoldPolish base error correction protocol. Mapped long reads are *k*-merized and inserted into an array of targeted Bloom filters with different *k*-mer sizes using ntHits[8]. Then, ntEdit[9] uses these Bloom filters to correct mismatches and small indels, and marks the regions it is unable to fix. Finally, Sealer[10] detects these unresolved regions as gaps and attempts to fill them using the targeted Bloom filters.

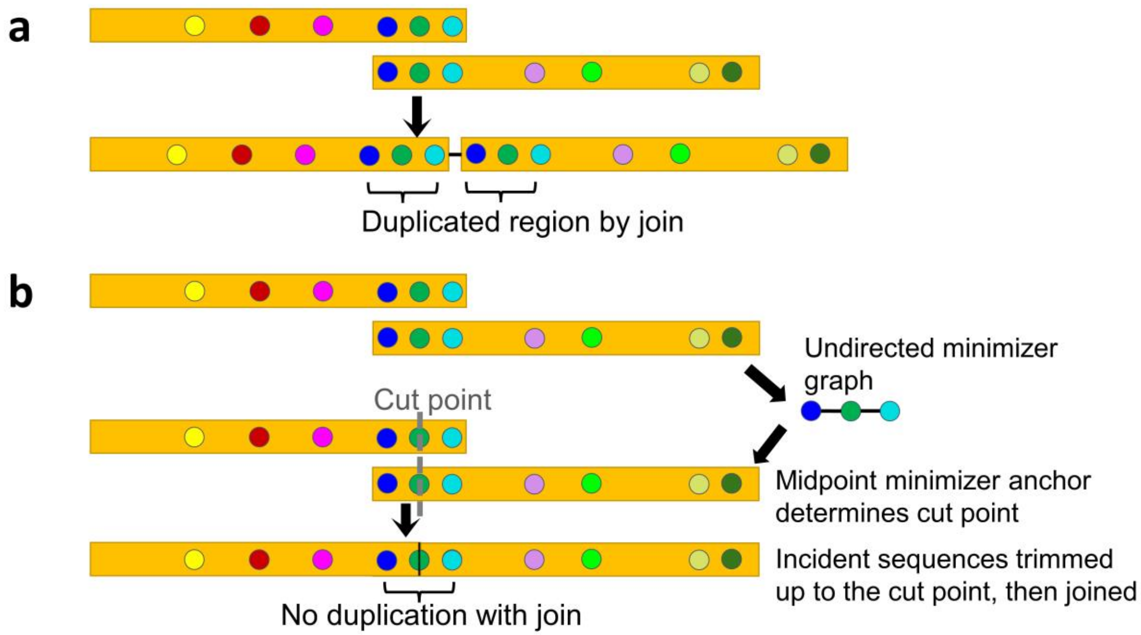

Supplementary Fig. 11: The overlap detection and resolution feature of ntLink. In these schematics, the circles represent minimizers, and the same colour indicates the same minimizer. **a** In previous versions of ntLink[11], overlapping sequences were joined end-to-end, which created small insertion misassemblies. **b** The overlap detection and resolution mode developed in ntLink detects and resolves these overlapping regions to avoid these spurious insertions. First, minimizers with a smaller *k* and *w* compared to the ntLink pairing stage are computed in the putative overlap region for a pair of sequences. The shared minimizers in this region with a multiplicity of one in each sequence (dark blue, medium green and light blue minimizers in **b**) are retained, and used to create an undirected minimizer graph. In this graph, the nodes are minimizers and adjacent minimizers are connected by edges. This graph is traversed to detect a mapping block between the sequence ends, and the position of the middle minimizer in the block chosen as the cut point. Finally, each sequence is trimmed up to the cut point and concatenated.

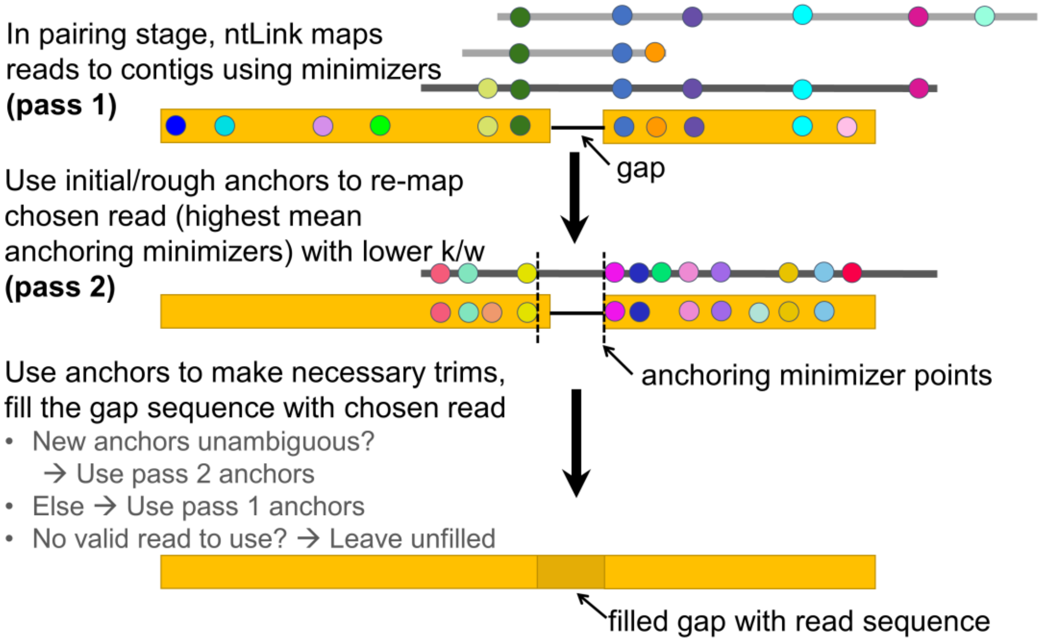

Supplementary Fig. 12: The gap-filling feature of ntLink. In filling a gap between a sequence pair, the read that supports the join with the highest mean number of anchoring minimizers is chosen as the representative sequence to fill the gap (pass 1 minimizers). The read is re-mapped to the incident sequences with a lower *k* and *w* for a more specific mapping (pass 2 minimizers). If the pass 2 minimizers are unambiguous, the anchoring minimizers are determined with these mappings, otherwise the pass 1 minimizers are used. These anchoring minimizers guide the trimming of the incident sequences as well as the chosen read for gap-filling. Finally, the trimmed sequences are concatenated together to output the scaffold with the filled gap.

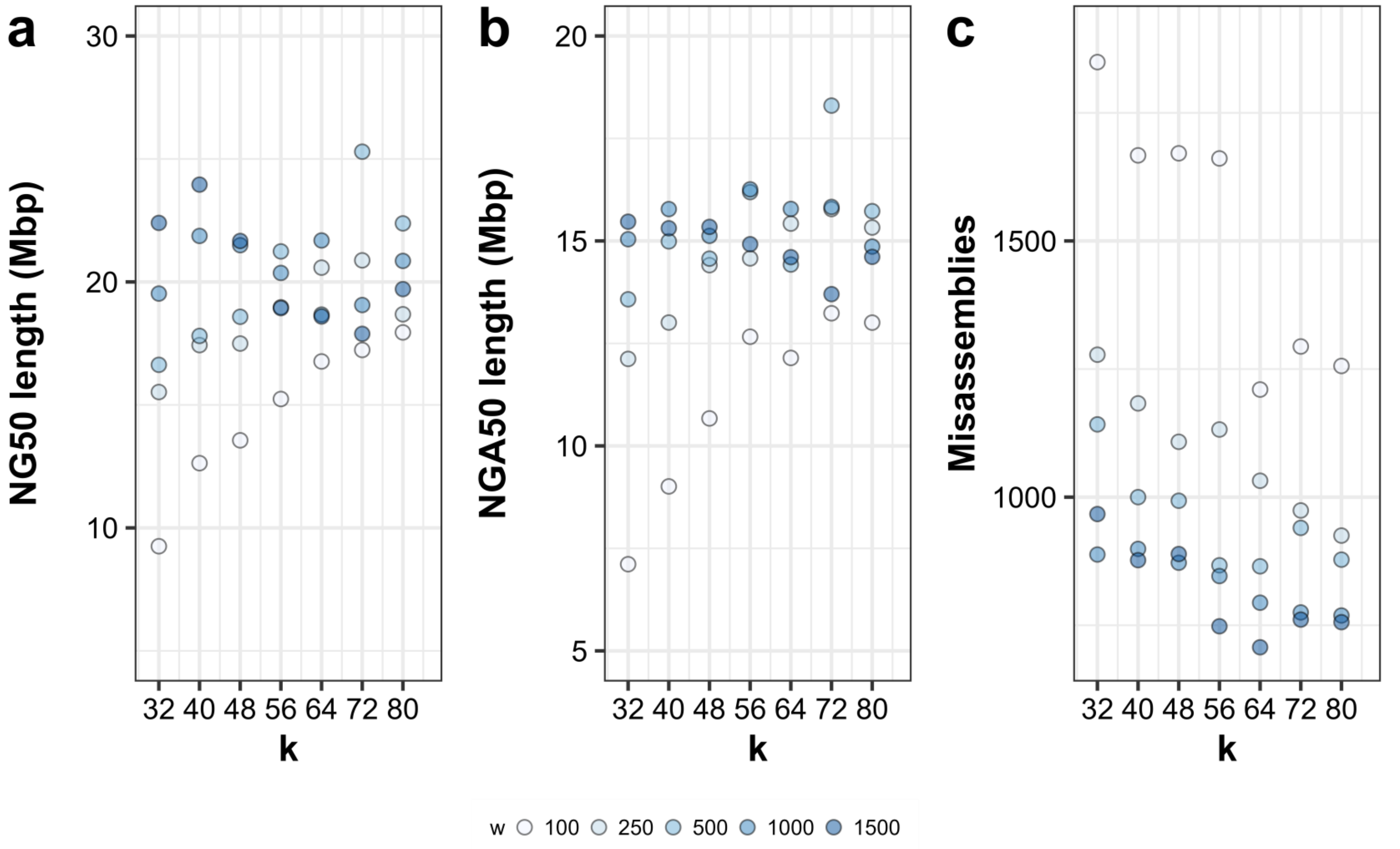

Supplementary Fig. 13: Contiguity and correctness results of assembling long reads from the human cell line NA24385 with GoldRush, sweeping on the GoldChain *k* and *w* parameters.

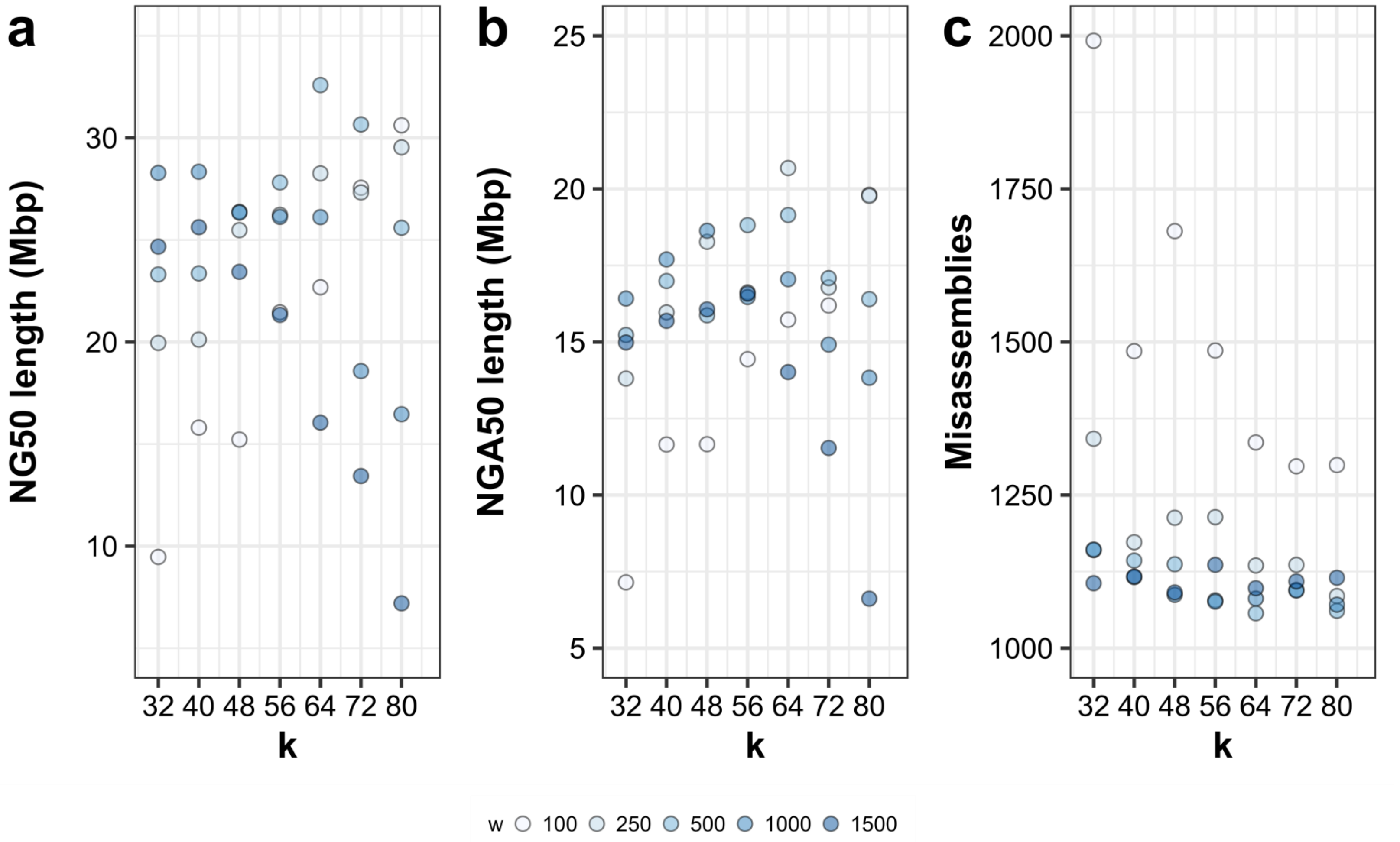

Supplementary Fig. 14: Contiguity and correctness results of assembling long reads from the human cell line HG01243 using GoldRush, sweeping on the GoldChain *k* and *w* parameters.

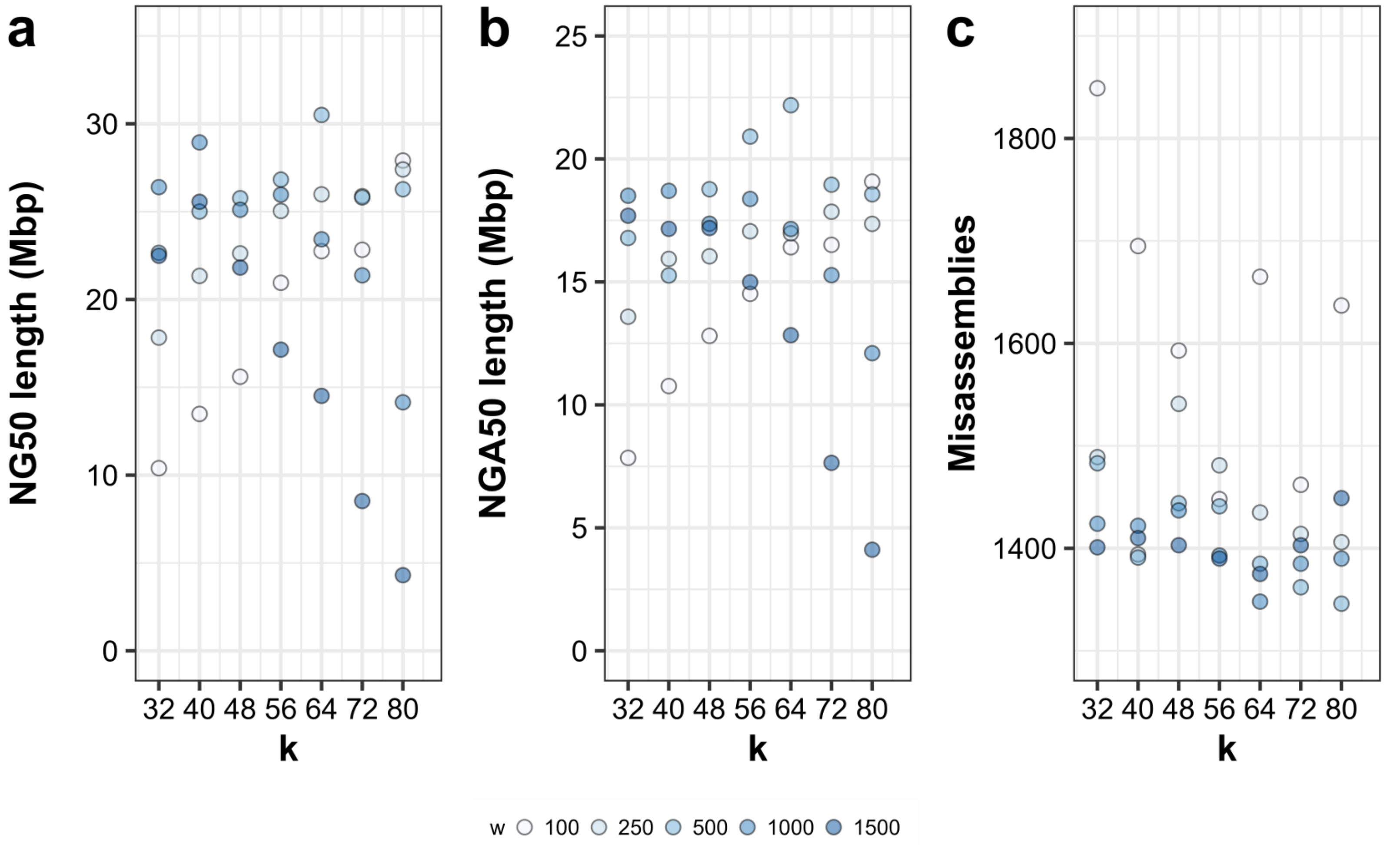

Supplementary Fig. 15: Contiguity and correctness results of assembling long reads from the human cell line HG02055 using GoldRush, sweeping on the GoldChain *k* and *w* parameters.

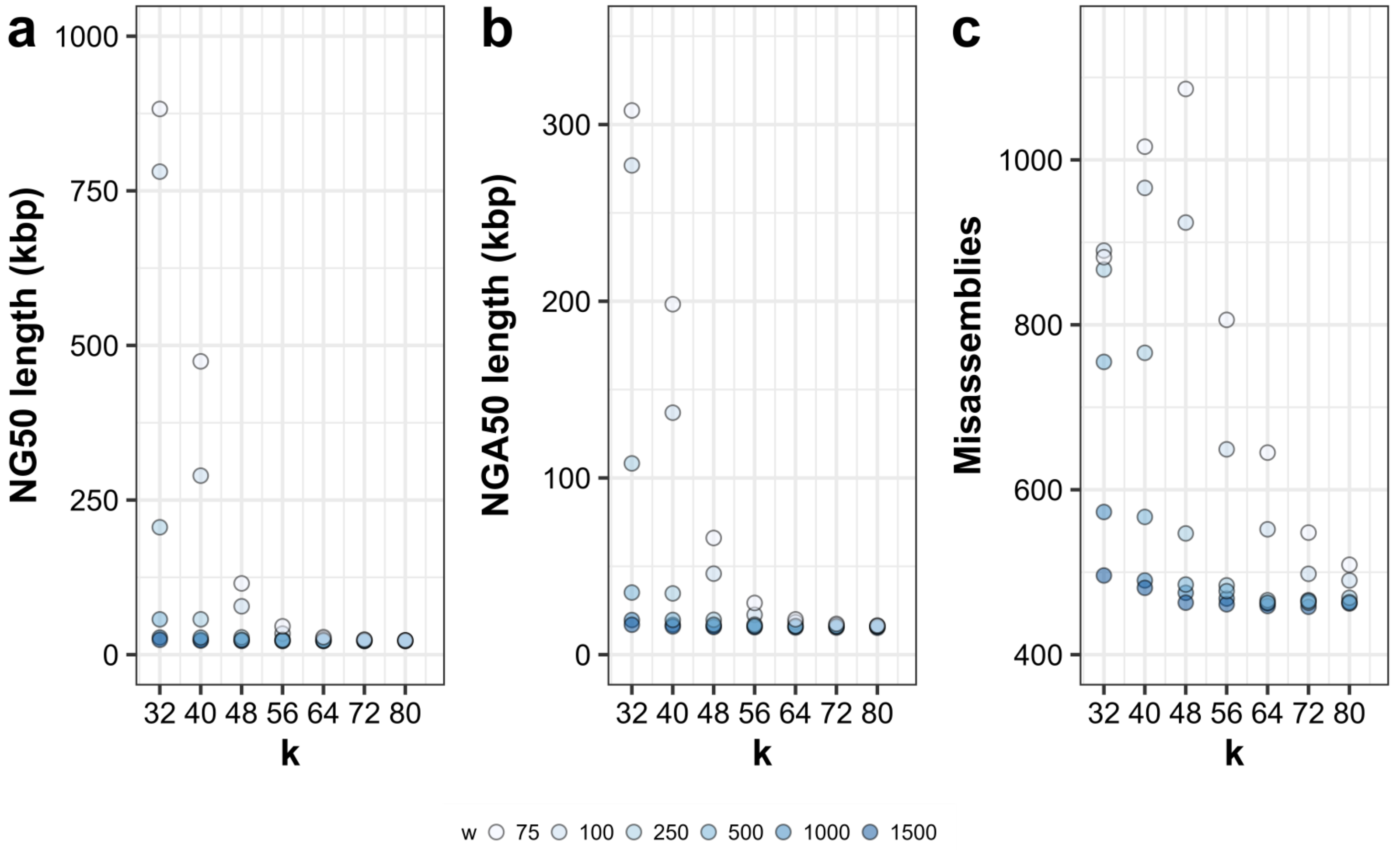

Supplementary Fig. 16: Contiguity and correctness results of assembling long reads from *O. sativa* using GoldRush, sweeping on the GoldChain *k* and *w* parameters.

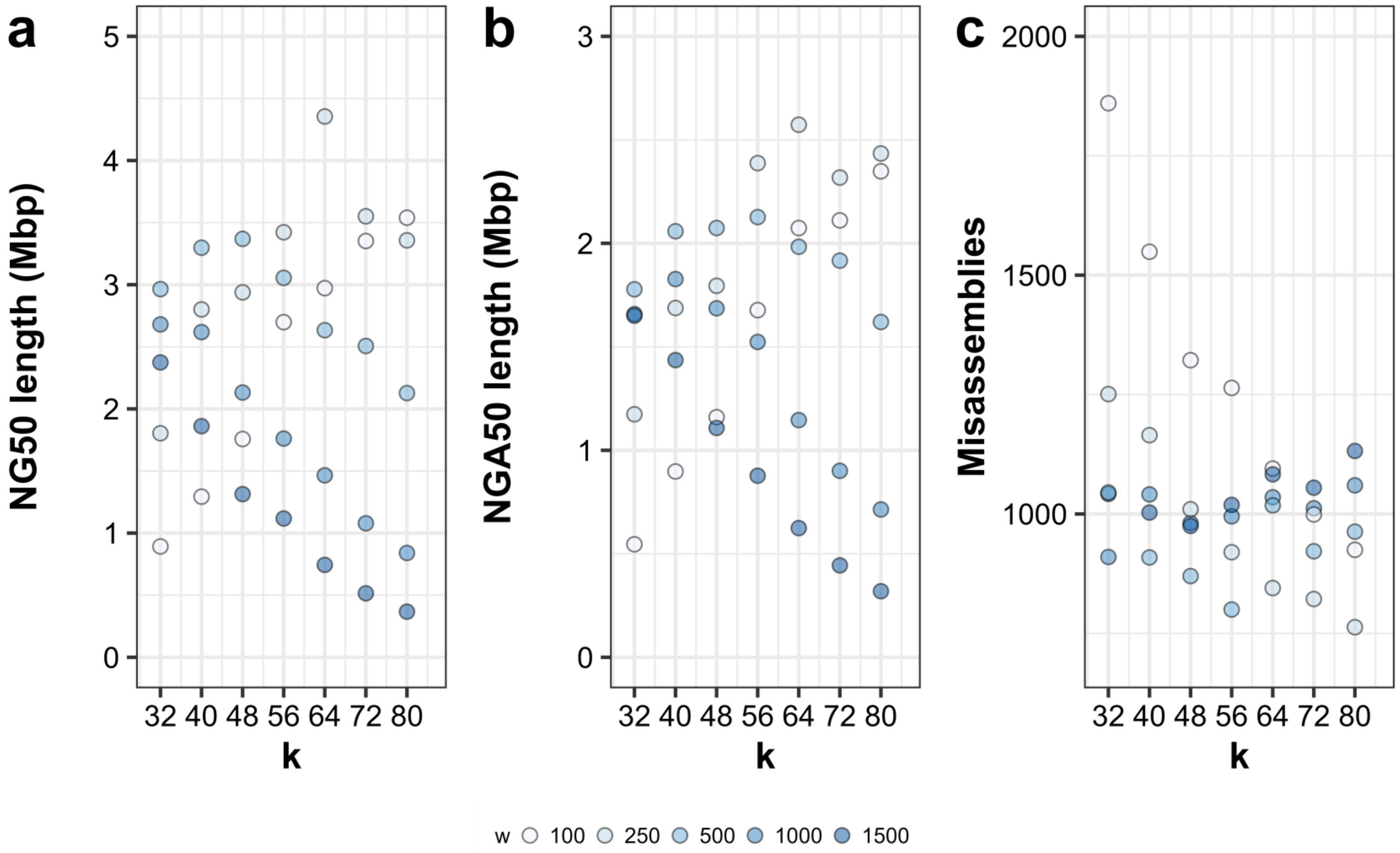

Supplementary Fig. 17: Contiguity and correctness results of assembling long reads from *S. lycopersicum* using GoldRush, sweeping on the GoldChain *k* and *w* parameters.

Supplementary Table 1: Contiguity and correctness statistics of human cell line NA24385 genome assemblies generated by GoldRush and the comparator tools. All statistics were generated using QUAST[12].

Assembler	Scaffold NG50 (Mbp)	Scaffold NGA50 (Mbp)	Contig NG50 (Mbp)	Contig NGA50 (Mbp)	Num. misassemblies	Num. local misassemblies	Genome fraction (%)	Total length (Gbp)	Duplication ratio	Num. mismatches per 100 kbp	Num. indels per 100 kbp	Num. N's per 100 kbp
**GoldRush**	25.3	18.3	21.6	16.3	940	1,210	94.7	2.9	1.0	257.2	227.1	53.7
**Flye**	26.6	26.1	26.6	26.1	940	1,679	96.3	2.9	1.0	140.6	54.5	0.0
**Redbean**	8.0	7.3	8.0	7.3	4,918	2,416	92.1	2.9	1.1	255.7	154.4	0.0
**Shasta**	29.7	24.1	29.7	24.1	1,682	2,242	95.8	2.9	1.0	150.8	57.0	0.0

Supplementary Table 2: Contiguity and correctness statistics of human cell line HG01243 genome assemblies generated by GoldRush and the comparator tools. All statistics were generated using QUAST[12].

Assembler	Scaffold NG50 (Mbp)	Scaffold NGA50 (Mbp)	Contig NG50 (Mbp)	Contig NGA50 (Mbp)	Num. misassemblies	Num. local misassemblies	Genome fraction (%)	Total length (Gbp)	Duplication ratio	Num. mismatches per 100 kbp	Num. indels per 100 kbp	Num. N's per 100 kbp
**GoldRush**	32.6	19.1	26.7	18.0	1,057	4,010	94.5	3.0	1.1	1,387.2	1,034.3	47.5
**Flye**	31.3	25.6	31.3	25.6	1,062	1,473	96.2	2.9	1.0	148.7	110.6	0
**Redbean**	10.3	8.2	10.3	8.2	7,052	2,267	92.8	2.9	1.1	324.7	354.5	0
**Shasta**	35.2	30.0	35.2	30.0	3,240	3,234	96.7	2.9	1.0	195.6	100.7	0

Supplementary Table 3: Contiguity and correctness statistics of human cell line HG02055 genome assemblies generated by GoldRush and the comparator tools. All statistics were generated using QUAST[12].

Assembler	Scaffold NG50 (Mbp)	Scaffold NGA50 (Mbp)	Contig NG50 (Mbp)	Contig NGA50 (Mbp)	Num. misassemblies	Num. local misassemblies	Genome fraction (%)	Total length (Gbp)	Duplication ratio	Num. mismatches per 100 kbp	Num. indels per 100 kbp	Num. N's per 100 kbp
**GoldRush**	30.5	22.2	28.2	17.3	1,385	4,667	94.7	3.1	1.1	1,985.0	1,415.5	57.1
**Flye**	38.8	30.4	38.8	30.4	1,048	1,697	96.4	2.9	1.0	155.5	126.0	0.0
**Redbean**	10.9	8.2	10.9	8.2	7,383	2,663	93.1	3.0	1.0	346.1	379.1	0.0
**Shasta**	39.6	33.7	39.6	33.7	3,314	3,217	96.6	2.9	1.0	210.2	103.9	0.0

Supplementary Table 4: Contiguity and correctness statistics of *O. sativa* genome assemblies generated by GoldRush and the comparator tools. All statistics were generated using QUAST[12].

Assembler	Scaffold NG50 (Mbp)	Scaffold NGA50 (Mbp)	Contig NG50 (Mbp)	Contig NGA50 (Mbp)	Num. misassemblies	Num. local misassemblies	Genome fraction (%)	Total length (Mbp)	Duplication ratio	Num. mismatches per 100 kbp	Num. indels per 100 kbp	Num. N's per 100 kbp
**GoldRush**	0.9	0.3	0.9	0.3	882	16,291	78.3	451.7	1.1	8,488.4	5,164.9	56.7
**Flye**	3.1	1.9	2.9	1.9	679	858	94.4	394.2	1.1	1,197.6	1,201.1	0.2
**Redbean**	5.2	2.4	5.2	2.4	483	1,663	90.0	363.2	1.0	1,344.4	2,386.5	0.0
**Shasta**	0.1	0.1	0.1	0.1	344	3,658	71.5	284.2	1.0	2,496.8	2,177.5	0.0

Supplementary Table 5: Contiguity and correctness statistics of *S. lycopersicum* genome assemblies generated by GoldRush and the comparator tools. All statistics were generated using QUAST[12].

Assembler	Scaffold NG50 (Mbp)	Scaffold NGA50 (Mbp)	Contig NG50 (Mbp)	Contig NGA50 (Mbp)	Num. misassemblies	Num. local misassemblies	Genome fraction (%)	Total length (Gbp)	Duplication ratio	Num. mismatches per 100 kbp	Num. indels per 100 kbp	Num. N's per 100 kbp
**GoldRush**	4.4	2.6	3.9	2.5	845	4,161	90.5	1.0	1.2	4,180.8	2,052.1	96.4
**Flye**	11.2	9.8	10.5	9.8	132	244	97.1	0.8	1.0	0.2	537.5	207.4
**Redbean**	0.3	0.2	0.3	0.2	4,895	13,726	94.8	2.2	2.7	0	6,283.2	5,633.5
**Shasta**	26.9	21.6	26.9	21.6	82	1,062	97.0	0.8	1.0	0	468.7	487.1

Supplementary Table 6: ONT long read sequencing reads used for genome assembly benchmarks. All datasets were sequenced using the R9.4.1 chemistry except for the *O. sativa* dataset, which is unknown. The error rate of each dataset was estimated using NanoSim[13]. The *S. lycopersicum* dataset was subsampled to simulate the typical sequencing depth needed for genome assembly.

Species	Cell line/Strain	Fold Coverage	N50 Length (bp)	Accession(s)/ Source	Basecaller	Estimated Error rate (%)
H. sapiens	NA24385	67	30,348	s3://ont-open-data/gm24385_2020.11/analysis/r9.4.1/20201026_1644_2-E5-H5_PAG07162_d7f262d5/guppy_v4.0.11_r9.4.1_hac_prom/align_unfiltered/chr1/guppy_v5.0.6_r9.4.1_sup_prom/	Guppy v5	4
H. sapiens	HG01243	63	45,310	https://s3-us-west-2.amazonaws.com/human-pangenomics/index.html?prefix=NHGRI_UCSC_panel/HG01243/nanopore/Guppy_4.2.2/	Guppy v4	9
H. sapiens	HG02055	71	47,934	https://s3-us-west-2.amazonaws.com/human-pangenomics/index.html?prefix=NHGRI_UCSC_panel/HG02055/nanopore/Guppy_4.2.2/	Guppy v4	11
O. sativa	Japonica group	62	29,349	SRR10589512-SRR10589711	-	20
S. lycopersicum	MoneybergTMV cultivar	72	41,999	ERR6668574, ERR7928672	Guppy v5	8

"-" denotes unknown.

Supplementary Table 7: Resource usage of GoldRush and the comparator tools for the genome assembly of the human cell line NA24385. Each genome assembler was run using 48 threads. Time (wall clock) and peak memory usage were recorded using the unix *time* command.

Assembler	Time (h)	Peak memory (GB)
**GoldRush**	16.6	51.9
**Flye**	33.7	502.4
**Redbean**	53.3	335.1
**Shasta**	4.4	1,003.0

Supplementary Table 8: Resource usage of GoldRush and the comparator tools for the genome assembly of the human cell line HG01243. Each genome assembler was run using 48 threads. Time (wall clock) and peak memory usage were recorded using the unix *time* command.

Assembler	Time (h)	Peak memory (GB)
**GoldRush**	20.8	53.9
**Flye**	47.5	366.4
**Redbean**	68.0	329.3
**Shasta**	4.1	884.8

Supplementary Table 9: Resource usage of GoldRush and the comparator tools for the genome assembly of the human cell line HG02055. Each genome assembler was run using 48 threads. Time (wall clock) and peak memory usage were recorded using the unix *time* command.

Assembler	Time (h)	Peak memory (GB)
**GoldRush**	20.8	54.5
**Flye**	49.2	441.0
**Redbean**	68.1	332.9
**Shasta**	5.0	1009.2

Supplementary Table 10: Resource usage of GoldRush and the comparator tools for the genome assembly of the *O. sativa* dataset. Each genome assembler was run using 48 threads. Time (wall clock) and peak memory usage were recorded using the unix *time* command.

Assembler	Time (h)	Peak memory (GB)
**GoldRush**	1.6	35.7
**Flye**	12.1	106.3
**Redbean**	1.4	67.8
**Shasta**	0.8	93.4

Supplementary Table 11: Resource usage of GoldRush and the comparator tools for the genome assembly of the *S. lycopersicum* dataset. Each stage was run using 48 threads. Time (wall clock) and peak memory usage were recorded using the unix *time* command.

Assembler	Time (h)	Peak memory (GB)
**GoldRush**	7.4	45.3
**Flye**	24.6	255.0
**Redbean**	14.1	160.6
**Shasta**	2.3	262.5

Supplementary Table 12: Contiguity and correctness statistics of human cell line NA24385 genome assembly generated by GoldRush at different stages. All statistics were generated using QUAST[12].

Stage	Scaffold NG50 (Mbp)	Scaffold NGA50 (Mbp)	Contig NG50 (Mbp)	Contig NGA50 (Mbp)	Num. misassemblies	Num. local misassemblies	Genome fraction (%)	Total length (Gbp)	Duplication ratio	Num. mismatches per 100 kbp	Num. indels per 100 kbp	Num. N's per 100 kbp
**GoldPath**	0.02	0.02	0.02	0.02	2,948	1,994	92.6	2.9	1.1	1,463.7	1,327.2	0.0
**GoldPolish**	0.02	0.02	0.02	0.02	2,990	1,777	92.7	2.9	1.1	228.5	197.2	23.4
**Tigmint-Long**	0.02	0.02	0.02	0.02	1,863	900	92.6	2.9	1.1	224.1	196.1	23.2
**GoldChain**	25.3	18.3	21.6	16.3	940	1,210	94.7	2.9	1.0	257.2	227.1	53.7

Supplementary Table 13: BUSCO statistics for assemblies of human cell line NA24385 generated by GoldRush and the comparator tools. BUSCO was run using the primates_odb10 lineage and a total of 13,780 BUSCO groups were searched [14].

Assembler	Complete BUSCOs	Complete and single-copy BUSCOs	Complete and duplicated BUSCOs	Fragmented BUSCOs	Missing BUSCOs
**GoldRush**	12,272 (89.1%)	12,059	213	511	997
**Flye**	12,988 (94.3%)	12,769	219	260	532
**Redbean**	12,193 (88.5%)	11,967	226	412	1,175
**Shasta**	12,920 (93.8%)	12,703	217	199	661

Supplementary Table 14: Counts of duplicated genes found in multiple copies in genome assemblies of the NA24385 human cell line, generated by GoldRush and comparator tools. The asmgene utility in minimap2[1] was run using an identity of 0.90 and a coverage of 0.99 using all cDNA sequences in GRCh38 from Ensembl[2] (release 87). The T2T-CHM13[3] (v1.1) genome assembly was used as reference and contained 2,461 duplicated genes.

Assembler	Duplicated gene count
**GoldRush**	845
**Flye**	1,725
**Redbean**	680
**Shasta**	1,717

Supplementary Table 15: Contiguity and correctness statistics of human cell line HG01243 genome assembly generated by GoldRush at different stages. All statistics were generated using QUAST[12].

Stage	Scaffold NG50 (Mbp)	Scaffold NGA50 (Mbp)	Contig NG50 (Mbp)	Contig NGA50 (Mbp)	Num. misassemblies	Num. local misassemblies	Genome fraction (%)	Total length (Gbp)	Duplication ratio	Num. mismatches per 100 kbp	Num. indels per 100 kbp	Num. N's per 100 kbp
**GoldPath**	0.03	0.03	0.03	0.03	1,859	4,611	93.1	2.9	1.1	3,502.2	3,002.8	0.0
**GoldPolish**	0.04	0.03	0.04	0.03	1,849	4,554	93.2	2.9	1.1	1,372.3	980.1	45.4
**Tigmint-Long**	0.03	0.03	0.03	0.03	1,490	1,946	92.9	2.9	1.1	1,347.2	969.6	44.3
**GoldChain**	32.6	19.1	26.7	18.0	1,057	4,010	94.5	3.0	1.1	1,387.2	1,034.3	47.5

Supplementary Table 16: Contiguity and correctness statistics of human cell line HG02055 genome assembly generated by GoldRush at different stages. All statistics were generated using QUAST[12].

Stage	Scaffold NG50 (Mbp)	Scaffold NGA50 (Mbp)	Contig NG50 (Mbp)	Contig NGA50 (Mbp)	Num. misassemblies	Num. local misassemblies	Genome fraction (%)	Total length (Gbp)	Duplication ratio	Num. mismatches per 100 kbp	Num. indels per 100 kbp	Num. N's per 100 kbp
**GoldPath**	0.03	0.03	0.03	0.03	2,082	5,183	93.3	3.0	1.1	4,266.9	3,425.9	0.0
**GoldPolish**	0.03	0.03	0.03	0.03	2,076	5,216	93.4	3.0	1.1	1,981.7	1,354.8	48.4
**Tigmint-Long**	0.03	0.03	0.03	0.03	1,695	2,297	93.1	3.0	1.1	1,947.1	1,339.7	47.2
**GoldChain**	30.5	22.2	28.2	17.3	1,385	4,667	94.7	3.1	1.1	1,985.0	1,415.5	57.1

Supplementary Table 17: BUSCO statistics for assemblies of human cell line HG01243 generated by GoldRush and the comparator tools. BUSCO was run using the primates_odb10 lineage and a total of 13,780 BUSCO groups were searched [14].

Assembler	Complete BUSCOs	Complete and single-copy BUSCO	Complete and duplicated BUSCO	Fragmented BUSCO	Missing BUSCOs
**GoldRush**	8,413 (61.1%)	8,295	118	597	4,770
**Flye**	12,344 (89.6%)	12,130	214	526	910
**Redbean**	10,964 (79.6%)	10,789	175	725	2,091
**Shasta**	12,485 (90.6%)	12,279	206	464	831

Supplementary Table 18: BUSCO statistics for assemblies of human cell line HG02055 generated by GoldRush and the comparator tools. BUSCO was run using the primates_odb10 lineage and a total of 13,780 BUSCO groups were searched [14].

Assembler	Complete BUSCOs	Complete and single-copy BUSCOs	Complete and duplicated BUSCOs	Fragmented BUSCOs	Missing BUSCOs
**GoldRush**	7,108 (51.6%)	7,016	92	569	6,103
**Flye**	12,215 (88.6%)	11,985	230	559	1,006
**Redbean**	10,763 (78.1%)	10,592	171	669	2,348
**Shasta**	12,471 (90.5%)	12,261	210	451	858

Supplementary Table 19: Resource usage breakdown of each GoldRush stage for the genome assembly of human cell line NA24385 using Racon instead of GoldPolish. Each stage was run using 48 threads. Time (wall clock) and peak memory usage were recorded using the unix *time* command.

Stage	Time (h)	Peak memory (GB)
**GoldPath**	3.2	51.9
**Racon**	9.9	602.3
**Tigmint-Long**	0.9	11.2
**GoldChain**	4.0	41.9
**TOTAL GoldRush**	18.0	602.3

Supplementary Table 20: Resource usage breakdown of each GoldRush stage for the genome assembly of human cell line NA24385. Each stage was run using 48 threads. Time (wall clock) and peak memory usage were recorded using the unix *time* command.

Stage	Time (h)	Peak memory (GB)
**GoldPath**	3.2	51.9
**GoldPolish**	8.3	11.0
**Tigmint-Long**	1.0	11.4
**GoldChain**	4.1	42.9
**TOTAL GoldRush**	16.6	51.9

Supplementary Table 21: Contiguity and correctness statistics of the GoldRush genome assembly of the human cell line NA24385 using Racon instead of GoldPolish at different stages. All statistics were generated using QUAST[12].

Stage	Scaffold NG50 (Mbp)	Scaffold NGA50 (Mbp)	Contig NG50 (Mbp)	Contig NGA50 (Mbp)	Num. misassemblies	Num. local misassemblies	Genome fraction (%)	Total length (Gbp)	Duplication ratio	Num. mismatches per 100 kbp	Num. indels per 100 kbp	Num. N's per 100 kbp
**GoldPath**	0.02	0.02	0.02	0.02	2,948	1,994	92.6	2.9	1.1	1,463.7	1,327.2	0.0
**Racon**	0.02	0.02	0.02	0.02	2,750	479	92.8	2.9	1.1	157.0	106.4	0.0
**Tigmint-Long**	0.02	0.02	0.02	0.02	1,665	410	92.7	2.9	1.1	155.8	105.9	0.0
**GoldChain**	24.4	14.9	24.0	14.4	945	589	94.6	2.9	1.0	184.9	134.2	33.8

Supplementary Table 22: BUSCO statistics for assemblies of human cell line NA24385 generated by GoldRush using Racon instead of GoldPolish. BUSCO was run using the primates_odb10 lineage and a total of 13,780 BUSCO groups were searched [14].

Complete BUSCOs	Complete and single-copy BUSCOs	Complete and duplicated BUSCOs	Fragmented BUSCOs	Missing BUSCOs
12,752 (92.5%)	12,533	219	296	732

Supplementary Table 23: Reference-free QV estimation statistics using Merqury for NA24385 GoldRush genome assemblies with GoldPolish or Racon. Merqury[4] was run using a *k*-mer database generated by Meryl[4] using *k* = 21 with NA24835 Illumina short reads (SRR11321732) from the Genome in a Bottle Consortium[5].

Polisher	Number of *k*-mers unique to assembly	Number of *k*-mers in read and assembly	QV	Error rate
**GoldPolish**	80,156,053 (2.7%)	2,876,045,151 (97.3%)	28.7	0.0013
**Racon**	46,770,784 (1.6%)	2,875,548,153 (98.4%)	31.1	0.0008

Supplementary Table 24: Reference-free QV estimation statistics using Merqury for non-repetitive or repetitive regions of the NA24385 GoldRush genome assembly using GoldPolish for the polishing step. Merqury[4] was run on a *k*-mer database generated by Meryl[4] using *k* = 21 with NA24835 Illumina short reads (SRR11321732) from the Genome in a Bottle Consortium[5].

Genomic region	Number of *k*-mers unique to assembly	Number of *k*-mers in read and assembly	QV	Error rate
**Non-repetitive**	47,412,119 (3.3%)	1,352,759,850 (96.6%)	27.7	0.0017
**Repetitive**	28,503,367 (2.0%)	1,386,439,261 (98.0%)	30.0	0.0010

Supplementary Table 25: Reference-free QV estimation statistics using Merqury for non-repetitive or repetitive regions of the NA24385 GoldRush genome assembly using Racon for the polishing step. Merqury[4] was run on a *k*-mer database generated by Meryl[4] using *k* = 21 with NA24835 Illumina short reads (SRR11321732) from the Genome in a Bottle Consortium[5].

Genomic region	Number of *k*-mers unique to assembly	Number of *k*-mers in read and assembly	QV	Error rate
**Non-repetitive**	26,372,962 (1.9%)	1,350,260,808 (98.1%)	30.3	0.0009
**Repetitive**	18,031,234 (1.3%)	1,387,849,704 (98.7%)	32.1	0.0006

Supplementary Table 26: Resource usage breakdown of each GoldRush stage for the genome assembly of the human cell line HG01243. Each stage was run using 48 threads. Time (wall clock) and peak memory usage were recorded using the unix *time* command.

Stage	Time (h)	Peak memory (GB)
**GoldPath**	4.3	53.9
**GoldPolish**	12.0	12.3
**Tigmint-Long**	0.8	11.1
**GoldChain**	3.7	26.3
**TOTAL GoldRush**	20.8	53.9

Supplementary Table 27: Resource usage breakdown of each GoldRush stage for the genome assembly of the human cell line HG02055. Each stage was run using 48 threads. Time (wall clock) and peak memory usage were recorded using the unix *time* command.

Stage	Time (h)	Peak memory (GB)
**GoldPath**	3.6	54.5
**GoldPolish**	12.5	13.3
**Tigmint-Long**	0.9	11.7
**GoldChain**	4.0	25.8
**TOTAL GoldRush**	21.0	54.5

Supplementary Table 28: Run time (wall clock) breakdown of each ntLink round within the GoldChain stage of GoldRush, NA24385 genome assembly. Time (wall clock) was recorded using the unix *time* command.

GoldChain Round	Time (h)
**1**	1.6
**2**	0.7
**3**	0.6
**4**	0.6
**5**	0.6
**TOTAL GoldChain**	4.0

Supplementary Table 29: Optimized parameters used for the GoldRush genome assemblies. Default parameters were used for GoldRush parameters not listed.

GoldRush Assembly	Optimized Parameters
**NA24385**	*G*=3e9 *k_ntLink*=72 *w_ntLink*=500
**HG01243**	*G*=3e9 *k_ntLink*=64 *w_ntLink*=500
**HG02055**	*G*=3e9 *k_ntLink*=64 *w_ntLink*=500
O. sativa	*G*=373e6 *k_ntLink*=32 *w_ntLink*=75
S. lycopersicum	*G*=824e6 *k_ntLink*=80

Supplementary Table 30: **Reference genome builds used for QUAST assembly analysis.**

Species	Reference genome build	Reference genome accession
H. sapiens	GRCh38	GCA_000001405.15
O. sativa	IRGSP-1.0	GCF_001433935.1
S. lycopersicum	MbTMVv1	GCA_915070445.1

Supplementary Table 31: **NA24385 cell line data used for Merqury base quality analysis.**

Data	Accession
**Short reads**	SRR11321732
**Maternal reference-grade genome assembly**	GCA_021951015.1
**Paternal reference-grade genome assembly**	GCA_021950905.1

Supplementary Table 32: **Reference genome used for gene duplication analysis.**

Species	Reference genome build	Reference genome accession
H. sapiens	T2T-CHM13v1.1	GCA_009914755.3

Supplementary Table 33: **cDNA sequences used for gene duplication analysis.**

Database	Release	Link
**Ensembl**	87	https://ftp.ensembl.org/pub/release-87/fasta/homo_sapiens/cdna/Homo_sapiens.GRCh38.cdna.all.fa.gz

Supplementary Table 34: Run time (wall clock) breakdown for each step of the first round of ntLink within the GoldChain stage of GoldRush, NA24385 genome assembly. Time (wall clock) was recorded using log statements from ntLink.

Step	Step description	Reading through reads file?	Time (min)
**pairing**	Mapping long reads to goldtigs, inferring goldtig pairs and outputting scaffold graph	Yes	41.4
**scaffolding**	Traversing scaffold graph to output goldtig paths	No	12.4
**overlap**	Resolving overlaps between adjacent goldtigs in paths	No	25.2
**gap-filling**	Filling scaffold gaps with read sequence	Yes	15.9
**TOTAL GoldChain round 1**			94.8 (1.6h)

Supplementary Method 1: **Improving the accuracy of the best hits for tiles.**
After the preliminary hits are established, the tiles are sequentially re-analyzed. The ID of the current tile is compared to the ID of the previous tile, and if the IDs do not match, the ID of the previous tile is queried against the current tile's ID-to-counts table. If the ID of the previous tile is found in the current tile's ID-to-counts table, the current tile's associated ID will be changed to that ID and the assignment will be adjusted depending on whether the newly changed ID has a count greater than the threshold (Supplementary Fig. 8a). Next, all the unassigned tiles in the read will be targeted. These tiles will be compared against their two adjacent tiles to see if the adjacent tiles share the same ID or have an ID that is 1 greater or smaller. If the adjacent tiles have the expected associated ID and these tiles are assigned, the current unassigned tile will be assigned (Supplementary Fig. 8b). After single unassigned tiles flanked by two assigned tiles are resolved, stretches of unassigned tiles flank by two assigned tiles will be targeted. If the assigned tiles flanking the unassigned region have the same ID or an ID that is 1 greater or smaller than the other, all the unassigned tiles that are flanked by the two tiles will be assigned and given the associated ID of either flank (Supplementary Fig. 8c). Finally, isolated assigned tiles are selected, and their assignment are changed to unassigned (Supplementary Fig. 8d).

Supplementary Note 1: **Time complexity analysis of GoldRush.**
GoldPath iterates through the read set to generate *M* (default 5) silver paths. The entire read set is first traversed to generated the Bloom filter used in the multi-index Bloom filter, then depending on the input sequencing coverage, the entire read set may be traversed again to generate the silver paths. Insertion and querying of spaced seeds in the multi-index Bloom filter[7] is completed in O(1) time. For a read of length *L*, there are *c* processing steps (described in Supplementary Figs. 6-9 and Supplementary Method 1), each of which takes O(*L*) time to complete. As both *c* and *L* << *n*, this step has a time complexity of O(*n*), where *n* is the number of reads.

Only the mapping stage of GoldPolish, minimap2[1], iterates through the reads to map to the golden path, henceforth referred to as the baseline assembly (length = *b*). minimap2 indexes the baseline assembly into minimizers and inserts them into hash data structures, which is created in O(*b*) time. A long read will then be mapped to the baseline assembly by querying the index, a constant O(1) operation, and chained using dynamic programming, which has a time complexity quadratic to the number of seeds (*s*). As both s^2 and *b* << *n*, the mapping stage in GoldPolish has a time complexity linear in the number of reads.

Similar to GoldPolish, only minimap2[1] in the mapping stage of Tigmint[11,15] iterates through the reads to map to the polished baseline assembly, and thus also has a time complexity linear in the number of reads.

Only two steps in GoldChain require passing through the entire long read dataset: the pairing stage and the gap-filling stage (Supplementary Table 34). In the pairing stage, the long reads are mapped to the baseline assembly using minimizer sketches. The assembly index hash data structure is created using the generated minimizers of sequences in O(*b*) time. Only unique minimizers in the baseline assembly are kept, ensuring that each minimizer corresponds to a single contig. Then, the minimizers for each long read are generated and queried against the index, which is completed in constant (O(1)) time. Therefore, mapping the reads against the sequences and outputting the inferred pairs in a scaffold graph format has O(*n*) time complexity overall, as *b* << *n*. Additional information about the mapping stage of this step can be found in the LongStitch publication[11]. For gap-filling, choosing which read to incorporate into the gap requires choosing a representative read, and the number of alternatives would be at most the maximum edge weight from the scaffold graph (*e_weight_max*) for a given gap. The read set must also be iterated through to collect the chosen reads for gap-filling. So, overall, this step has O(*b*×*e_weight_max* + *n*) time complexity. Given that the edge weight would be expected to be less than the read coverage (which could be considered a large constant here, given the reliance on unique minimizers in the draft sequences in creating the edges), and *b* << *n*, the *n* term would dominate. Therefore, all the steps for GoldChain are linear in the number of reads. Note that given the liftover-round functionality in the ntLink tool included in the GoldChain step, mapping the reads to the goldtigs is only required in the first scaffolding round.

Supplementary References

1. Li, H. Minimap2: pairwise alignment for nucleotide sequences. *Bioinformatics* **34**, 3094–3100 (2018).

2. Cunningham, F. *et al.* Ensembl 2022. *Nucleic Acids Res* **50**, D988–D995 (2022).

3. Nurk, S. *et al.* The complete sequence of a human genome. *Science (1979)* **376**, 44–53 (2022).

4. Rhie, A., Walenz, B. P., Koren, S. & Phillippy, A. M. Merqury: reference-free quality, completeness, and phasing assessment for genome assemblies. *Genome Biol* **21**, 245 (2020).

5. Zook, J. M. *et al.* Extensive sequencing of seven human genomes to characterize benchmark reference materials. *Sci Data* **3**, 160025 (2016).

6. Li, J. X., Coombe, L., Wong, J., Birol, I. & Warren, R. L. ntEdit+Sealer: Efficient Targeted Error Resolution and Automated Finishing of Long-Read Genome Assemblies. *Curr Protoc* **2**, e442 (2022).

7. Chu, J. *et al.* Mismatch-tolerant, alignment-free sequence classification using multiple spaced seeds and multiindex Bloom filters. *Proceedings of the National Academy of Sciences* **117**, 16961–16968 (2020).

8. Mohamadi, H., Chu, J., Coombe, L., Warren, R. & Birol, I. ntHits: *de novo* repeat identification of genomics data using a streaming approach. *bioRxiv* 2020.11.02.365809 (2020) doi:10.1101/2020.11.02.365809.

9. Warren, R. L. *et al.* ntEdit: scalable genome sequence polishing. *Bioinformatics* **35**, 4430–4432 (2019).

10. Paulino, D. *et al.* Sealer: a scalable gap-closing application for finishing draft genomes. *BMC Bioinformatics* **16**, 230 (2015).

11. Coombe, L. *et al.* LongStitch: high-quality genome assembly correction and scaffolding using long reads. *BMC Bioinformatics* **22**, 534 (2021).

12. Mikheenko, A., Prjibelski, A., Saveliev, V., Antipov, D. & Gurevich, A. Versatile genome assembly evaluation with QUAST-LG. *Bioinformatics* **34**, i142–i150 (2018).

13. Yang, C., Chu, J., Warren, R. L. & Birol, I. NanoSim: nanopore sequence read simulator based on statistical characterization. *Gigascience* **6**, gix010 (2017).

14. Simão, F. A., Waterhouse, R. M., Ioannidis, P., Kriventseva, E. v & Zdobnov, E. M. BUSCO: assessing genome assembly and annotation completeness with single-copy orthologs. *Bioinformatics* **31**, 3210–3212 (2015).

15. Jackman, S. D. *et al.* Tigmint: correcting assembly errors using linked reads from large molecules. *BMC Bioinformatics* **19**, 393 (2018).
