# Supplementary material for: Linear time complexity de novo long read genome assembly with GoldRush
Source: Nat Commun. 2023 May 22;14:2906. doi: 10.1038/s41467-023-38716-x (PMC10202940; doi:10.1038/s41467-023-38716-x)
Supplement: Supplementary file 3 — Reporting Summary [file 41467_2023_38716_MOESM3_ESM.pdf]

nature portfolio

Corresponding author(s): Johnathan Wong, Inanç Birol

Last updated by author(s): May 4, 2023

Reporting Summary

Nature Portfolio wishes to improve the reproducibility of the work that we publish. This form provides structure for consistency and transparency in reporting. For further information on Nature Portfolio policies, see our Editorial Policies and the Editorial Policy Checklist.

Statistics

For all statistical analyses, confirm that the following items are present in the figure legend, table legend, main text, or Methods section.

n/a	Confirmed	
☐	☒	The exact sample size (*n*) for each experimental group/condition, given as a discrete number and unit of measurement
☒	☐	A statement on whether measurements were taken from distinct samples or whether the same sample was measured repeatedly
☒	☐	The statistical test(s) used AND whether they are one- or two-sided<br>*Only common tests should be described solely by name; describe more complex techniques in the Methods section.*
☒	☐	A description of all covariates tested
☒	☐	A description of any assumptions or corrections, such as tests of normality and adjustment for multiple comparisons
☐	☒	A full description of the statistical parameters including central tendency (e.g. means) or other basic estimates (e.g. regression coefficient) AND variation (e.g. standard deviation) or associated estimates of uncertainty (e.g. confidence intervals)
☒	☐	For null hypothesis testing, the test statistic (e.g. *F*, *t*, *r*) with confidence intervals, effect sizes, degrees of freedom and *P* value noted<br>*Give P values as exact values whenever suitable.*
☒	☐	For Bayesian analysis, information on the choice of priors and Markov chain Monte Carlo settings
☒	☐	For hierarchical and complex designs, identification of the appropriate level for tests and full reporting of outcomes
☒	☐	Estimates of effect sizes (e.g. Cohen's *d*, Pearson's *r*), indicating how they were calculated

Our web collection on statistics for biologists contains articles on many of the points above.

Software and code

Policy information about availability of computer code

Data collection	GoldRush v1.0.0 (https://github.com/bcgsc/goldrush). de novo long read genome assembler.<br>Flye v2.9 (https://github.com/fenderglass/Flye). de novo long read genome assembler.<br>Redbean v2.5 (https://github.com/ruanjue/wtdbg2). de novo long read genome assembler.<br>Shasta v.0.10.0 (https://github.com/chanzuckerberg/shasta). de novo long read genome assembler.<br>Racon v1.5.0 (https://github.com/isovic/racon). Tool for polishing DNA sequences.<br>python v3.9.13. Language used to power GoldChain.
Data analysis	QUAST v5.0.2 (https://github.com/ablab/quast). Tool for assessing genome assembly contiguity and correctness.<br>BUSCO v5.3.2 (https://gitlab.com/ezlab/busco). Tool for assessing genome assembly completeness.<br>Merqury v1.3.0 (https://github.com/marbl/merqury). Tool for assessing genome assembly base quality<br>asmgene (Minimap2 v2.24) (https://github.com/lh3/minimap2). Tool for assessing gene duplication in genome assembly.<br>RepeatMasker v4.1.2 (https://www.repeatmasker.org/). Tool for masking repetitive genomic regions.<br>R v3.6.1. Language used to generate Figs. 2 and 3, and Supplementary Figs. 2, 5, 13-17.<br>ggplot2 v3.4.2. R package used to generate Figs. 2 and 3, and Supplementary Figs. 2, 5, 13-17.

For manuscripts utilizing custom algorithms or software that are central to the research but not yet described in published literature, software must be made available to editors and reviewers. We strongly encourage code deposition in a community repository (e.g. GitHub). See the Nature Portfolio guidelines for submitting code & software for further information.

Data

Policy information about availability of data

All manuscripts must include a data availability statement. This statement should provide the following information, where applicable:

- Accession codes, unique identifiers, or web links for publicly available datasets
- A description of any restrictions on data availability
- For clinical datasets or third party data, please ensure that the statement adheres to our policy

The GoldRush, Flye, Redbean, and Shasta genome assemblies generated in this study have been deposited in Zenodo at https://doi.org/10.5281/zenodo.7884681. The GoldRush genome assemblies generated for the parameter sweep experiments in Supplementary Figs. 13-17 are available upon request. The accession codes or location of sequencing data used for assembling the draft genomes are listed in Supplementary Table 6. The accession codes of the reference genomes and the short read dataset used to benchmark GoldRush and comparators' genome assemblies are provided in Supplementary Tables 30-33.

Human research participants

Policy information about studies involving human research participants and Sex and Gender in Research.

Reporting on sex and gender	This information has not been collected. No human participants were recruited for this study, therefore collection of gender information is not applicable.
Population characteristics	See above.
Recruitment	No human participants were recruited.
Ethics oversight	No ethics oversight is needed.

Note that full information on the approval of the study protocol must also be provided in the manuscript.

Field-specific reporting

Please select the one below that is the best fit for your research. If you are not sure, read the appropriate sections before making your selection.

☒ Life sciences ☐ Behavioural & social sciences ☐ Ecological, evolutionary & environmental sciences

For a reference copy of the document with all sections, see nature.com/documents/nr-reporting-summary-flat.pdf

Life sciences study design

All studies must disclose on these points even when the disclosure is negative.

Sample size	Each cell line (genomic long read dataset) had a sample size of 1. Genome assemblers, by design, take in sequenced reads from a single individual to assemble that individual's genome. To demonstrate GoldRush's robustness, we assembled 5 draft genomes, 3 of which are derived from different human individuals. The remaining 2 are from different plant species (rice and tomato).
Data exclusions	No data point generated from analyses were excluded.
Replication	No replication analyses were done. While the de novo long read genome assemblers are not all deterministic, they are intended to produce similar results between runs on the same data. QUAST, BUSCO, asmgene (minimap2), and Merqury analyses are deterministic.
Randomization	Allocation was not random. There were no group allocations so randomization is not relevant.
Blinding	No blinding was done. There were no group allocations so blinding is not relevant.

Reporting for specific materials, systems and methods

We require information from authors about some types of materials, experimental systems and methods used in many studies. Here, indicate whether each material, system or method listed is relevant to your study. If you are not sure if a list item applies to your research, read the appropriate section before selecting a response.

Materials & experimental systems

n/a	Involved in the study
☒	☐ Antibodies
☒	☐ Eukaryotic cell lines
☒	☐ Palaeontology and archaeology
☒	☐ Animals and other organisms
☒	☐ Clinical data
☒	☐ Dual use research of concern

Methods

n/a	Involved in the study
☒	☐ ChIP-seq
☒	☐ Flow cytometry
☒	☐ MRI-based neuroimaging
